# Supplementary material for: Comprehensive functional testing in fibroblasts has strong utility to diagnose mitochondrial disease
Source: EMBO Mol Med. 2026 Sep 8;18(10):4153–72. doi: 10.1038/s44321-026-00497-3 (PMC13658156; doi:10.1038/s44321-026-00497-3)
Supplement: Supplementary file 1 [file 44321_2026_497_MOESM4_ESM.pdf]

EMBO Molecular Medicine

Comprehensive functional testing in fibroblasts has strong utility to diagnose mitochondrial disease

Johan Van Hove, Marisa Friederich, Roxanne Van Hove, Jessica Lee, Kaz Knight, Tonia Donovan, Lori Silveira, Rebecca Ganetzky, Michio Hirano, Jose Abdenur, Merlin Butler, David Cassiman, Bruce Cohen, Sarah Elsea, Gregory M. Enns, William Gahl, Ralitza Gavrilova, Gabriella Geddes, Emma Glamuzima, Amy Goldstein, Richard Haas, Aneal Khan, Kimberly Kripps, Austin Larson, April Lehman, Uta Lichter-Konecki, Johannes Mayr, Eva Morava, James Peterson, Jill Rosenfeld, Russel Saneto, Fernando Scaglia, Emily Shelkowitz, Mariella Simon, Joél Smet, Wendy Smith, Claudia Soler-Alfonso, Mark Tarnopolsky, Rudy Van Coster, Arnaud Vanlander, Pieter Vermeersch, Gerard Vockley, Kristen Wigby, Lynne Wolfe, Saskia Wortmann, and Jennifer Yang

Corresponding authors: Johan Van Hove (johan.vanhove@childrenscolorado.org) , Marisa Friederich (marisa.friederich@cuanschutz.edu)

Review Timeline:

Submission Date:	8th Feb 26
Editorial Decision:	5th Mar 26
Revision Received:	12th Jun 26
Editorial Decision:	7th Jul 26
Revision Received:	15th Jul 26
Accepted:	22nd Jul 26

Editor: Zeljko Durdevic

Transaction Report:

(Note: With the exception of the correction of typographical or spelling errors that could be a source of ambiguity, letters and reports are not edited. Depending on transfer agreements, referee reports obtained elsewhere may or may not be included in this compilation. Referee reports are anonymous unless the Referee chooses to sign their reports.)

5th Mar 2026

Dear Dr. Van Hove,

Thank you for the submission of your manuscript to EMBO Molecular Medicine. We have now received feedback from the three reviewers who agreed to evaluate your manuscript. All three referees recognize interest of the study but also raise important concerns that should be addressed in a major revision. If you would like to discuss further the points raised by the referees, I am available to do so via email or video. Let me know if you are interested in this option.

Further consideration of a revision that addresses reviewers' concerns in full will entail a second round of review. EMBO Molecular Medicine encourages a single round of revision only and therefore, acceptance or rejection of the manuscript will depend on the completeness of your responses included in the next, final version of the manuscript. For this reason, and to save you from any frustrations in the end, I would strongly advise against returning an incomplete revision.

We would welcome the submission of a revised version within three months for further consideration. Please let us know if you require longer to complete the revision.

Please use this link to login to the manuscript system and submit your revision: https://embomolmed.msubmit.net/cgi-bin/main.plex

I look forward to receiving your revised manuscript.

Yours sincerely,

Zeljko Durdevic

Zeljko Durdevic
Senior Editor
EMBO Molecular Medicine

*****
When preparing your revised manuscript, please refer to our guidelines: https://link.springer.com/journal/44321/submission-guidelines#cms-Revised-submissions. We perform an initial quality control of all revised manuscripts before re-review; failure to include requested items will delay the evaluation of your revision.

We require:

1) A .docx formatted version of the manuscript text (including legends for main figures, EV figures and tables). Please make sure that the changes are highlighted to be clearly visible.

2) Individual production quality figure files as .eps, .tif, .jpg (one file per figure). For guidance, download the 'Figure Guide PDF': https://media.springernature.com/original/springer-cms/rest/v1/content/27825798/data/v1.

3) A .docx formatted letter INCLUDING the reviewers' reports and your detailed point-by-point responses to their comments. As part of the EMBO Press transparent editorial process, the point-by-point response is part of the Review Process File (RPF), which will be published alongside your paper.

4) A complete author checklist, which you can download from our author guidelines. Please insert information in the checklist that is also reflected in the manuscript. The completed author checklist will also be part of the RPF.

5) Please note that all corresponding authors are required to supply an ORCID ID for their name upon submission of a revised manuscript.

6) It is mandatory to include a 'Data Availability' section after the Materials and Methods. Before submitting your revision, primary datasets produced in this study need to be deposited in an appropriate public database, and the accession numbers and database listed under 'Data Availability'. Please remember to provide a reviewer password if the datasets are not yet public.

In case you have no data that requires deposition in a public database, please state so in this section. Note that the Data Availability Section is restricted to new primary data that are part of this study.

7) For data quantification: please specify the name of the statistical test used to generate error bars and P values, the number (n) of independent experiments (specify technical or biological replicates) underlying each data point and the test used to calculate p-values in each figure legend. The figure legends should contain a basic description of n, P and the test applied. Graphs must include a description of the bars and the error bars (s.d., s.e.m.).

8) At EMBO Press we ask authors to provide source data for the main manuscript figures. You will receive a separate email with instructions for providing source data with your revised manuscript, including how to upload and organize the files.

9) Our journal encourages inclusion of *data citations in the reference list* to directly cite datasets that were re-used and obtained from public databases. Data citations in the article text are distinct from normal bibliographical citations and should directly link to the database records from which the data can be accessed. In the main text, data citations are formatted as follows: "Data ref: Smith et al, 2001" or "Data ref: NCBI Sequence Read Archive PRJNA342805, 2017". In the Reference list, data citations must be labeled with "[DATASET]". A data reference must provide the database name, accession number/identifiers and a resolvable link to the landing page from which the data can be accessed at the end of the reference.

10) We replaced Supplementary Information with Expanded View (EV) Figures and Tables that are collapsible/expandable online. A maximum of 5 EV Figures can be typeset. EV Figures should be cited as 'Figure EV1, Figure EV2" etc... in the text and their respective legends should be included in the main text after the legends of regular figures.

- For the figures that you do NOT wish to display as Expanded View figures, they should be bundled together with their legends in a single PDF file called *Appendix*, which should start with a short Table of Content. Appendix figures should be referred to in the main text as: "Appendix Figure S1, Appendix Figure S2" etc.

- Additional Tables/Datasets should be labeled and referred to as Table EV1, Dataset EV1, etc. Legends have to be provided in a separate tab in case of .xls files. Alternatively, the legend can be supplied as a separate text file (README) and zipped together with the Table/Dataset file.

11) The paper explained: EMBO Molecular Medicine articles are accompanied by a summary of the articles to emphasize the major findings in the paper and their medical implications for the non-specialist reader. Please provide a draft summary of your article highlighting

- the medical issue you are addressing,

- the results obtained and

- their clinical impact.

This may be edited to ensure that readers understand the significance and context of the research. Please refer to any of our published articles for an example.

12) Author contributions: You will be asked to provide CRediT (Contributor Role Taxonomy) terms in the submission system. These replace a narrative author contribution section in the manuscript.

13) A Conflict of Interest statement should be provided in the main text.

14) Every published paper includes a 'Synopsis' to further enhance discoverability. Synopses are displayed on the journal webpage and are freely accessible to all readers. They include a short stand first (maximum of 300 characters, including space) as well as 2-5 one-sentences bullet points that summarizes the paper. Please write the bullet points to summarize the key NEW findings. They should be designed to be complementary to the abstract - i.e. not repeat the same text. We encourage inclusion of key acronyms and quantitative information (maximum of 30 words / bullet point). Please use the passive voice. Please attach these in a separate file or send them by email, we will incorporate them accordingly.

Please also suggest a striking image or visual abstract to illustrate your article as a PNG file 550 px wide x 300-600 px high.

15) Include a Reagents and Tools Table as part of the Methods section, which can be downloaded from our author guidelines.

EMBO Molecular Medicine has a "scooping protection" policy, whereby similar findings that are published by others during review or revision are not a criterion for rejection. Should you decide to submit a revised version, I do ask that you get in touch after three months if you have not completed it, to update us on the status.

***** Reviewer's comments *****

Referee #1 (Comments on Novelty/Model System for Author):

The reason for indicating "medium" medical impact rather than "high" is as follows. The Authors demonstrate that the battery of assays, either separately or combined, can provide mostly good to excellent sensitivity to diagnose primary mitochondrial of multiple origins. The idea would be for these assays, separately or combined, to be deployed when whole exome sequencing yields a novel variant in a nuclear DNA or mtDNA gene encoding a mitochondrial protein. This part of the study appears technically very well done, and the results are clear (though a little tedious to read, but the tabled information is clear), and potentially useful. My concern is that many/most of these assays are not easy to perform by non-experts in a sufficiently optimized way as to minimize false negatives. For examples, measuring enzyme activity of the electron transport chain complexes can result in values that are too low in controls, making it difficult to detect a decrease when one is present in a patient sample. As another example, BN-PAGE detection of assembled ETC complexes can require substantial optimization. Even if the methods were described in great detail (which they are not; only references are given), optimization in the aforementioned techniques would be needed. At the very least, the Authors should supply expected normal values (or a range), e.g. for enzyme activities, and provide a listing of references that contain what they judge as "excellent" normal values from labs that run the tests well. I think that this would easily improve the utility of this study.

Referee #1 (Remarks for Author):

The Authors demonstrate that a battery of assays, either separately or combined, can provide mostly good to excellent sensitivity to diagnose primary mitochondrial of multiple origins. The idea would be for these assays, separately or combined, to be deployed when whole exome sequencing yields a novel variant in a nuclear DNA or mtDNA gene encoding a mitochondrial protein. This part of the study appears technically very well done, and the results are clear (though a little tedious to read, but the tabled information is clear), and potentially useful.

Comments:
1. A major concern is that many/most of these assays are not easy to perform by non-experts (including experience researchers in the mitochondrial biology field who do not have experience with these particular approaches) in a sufficiently optimized way as to minimize false negatives. For examples, measuring enzyme activity of electron transport chain complexes can result in values that are too low in controls, making it difficult to detect a decrease when one is present in a patient sample. As another example, BN-PAGE detection of assembled ETC complexes can require substantial optimization. Even if the methods were described in great detail (which they are not; only references are given), optimization of the aforementioned techniques would be needed. At the very least, the Authors should supply - possibly in a table - expected normal values (or a range) where possible, e.g. for enzyme activities.

2. Continuing with the previous comment, Authors should provide references that contain what Authors' judge to be "excellent" normal values from labs that are deemed to run the tests well ("expert labs"). A list of such "expert" references can accompany each method, e.g., in a table. I think that this would be a relatively easy way to improve the utility of this study.

3. The Abstract should state that analyses were performed in skin fibroblasts

4. Should there be a recommendation as to the number of control samples to run for quantitative assays (e.g., enzyme activities)?

5. Should there be guidelines about culture conditions, especially substrate amounts (e.g., glucose concentration, inclusion or not of pyruvate) and confluence at the time of cell harvest?

6. In a few sentences, some rewording would improve clarity (e.g., in Discussion, para starting with "This study has two biases" - the second sentence (Cell lines with variants ...) could use some rewording).

Referee #2 (Remarks for Author):

This manuscript by Van Hove et al presents a broad-based biochemical approach to diagnosis of primary mitochondrial disease, which remains a significant challenge. The authors combine a variety of well-established biochemical approaches to present a compelling case for their proposed approach. The work captures the nuances involved in the diagnosis of a broad spectrum of

disease, ranging from respiratory chain enzyme defects to mtDNA maintenance disorders. A particular strength of the work is the use of >300 fibroblast cell lines, with multiple lines for the different subsets of mitochondrial diseases. The development of such a testing regimen is important for labs tasked with identifying and diagnosing mitochondrial diseases of unknown etiology. While the analyses presented are all standard assays that have "stood the test of time", thus being robust and rigorous enough to function in the diagnostic process, the data support the combined approach in primary mitochondrial disease diagnosis. This work is likely to be of great interest for clinicians and clinical diagnostic labs focusing on mitochondrial disease.

Minor comments:
The manuscript is clearly written and the conclusions drawn are supported by the data presented. There are, however, a couple of minor points that should be addressed:

1) Addressing some minor grammatical issues would improve manuscript flow i.e. abstract missing a couple of crucial commas.

2) It is disappointing that, in spite of the very large bibliography, there are no references to the seminal work done on mitochondrial disease diagnostics by the labs of Brian Robinson, Eric Shoubridge or Vamsi Mootha.

3) The use of antibiotics in the fibroblast cultures needs further explanation, given that some antibiotics have been shown to negatively affect mitochondrial function.

4) Cutoffs optimization: "...a gradual increase for complex IV....with no impact on complexes II and IV... " Did you mean Complex V?

Referee #3 (Comments on Novelty/Model System for Author):

The methodologies used are well established and solid. The study is not new, as fibroblasts and used assays have been used for decades, but the study may provide a general frame for the diagnosis of PMDs.

Referee #3 (Remarks for Author):

The manuscript by Van Hove and colleagues presents a comprehensive evaluation of functional mitochondrial assays performed in fibroblasts derived from a large cohort of patients with genetically confirmed primary mitochondrial disease (PMD), alongside disease controls and healthy controls. Fibroblasts from patients have been used as disease models for decades and have been an extremely useful tool to study the consequences of mutations. The authors systematically assess the diagnostic performance of respiratory chain enzyme activity measurements, blue native PAGE in-gel activity assays, complex I assembly analysis, and targeted protein abundance quantification. Overall, although not novel as methodology and outcome, the study addresses the very relevant diagnostic utility of functional validation in fibroblasts in the era of next-generation sequencing.
The is provides important quantitative evidence supporting the continued role of fibroblast-based functional assays in the diagnostic evaluation of mitochondrial disease. The manuscript is clearly written, the experimental approach is methodologically sound, and the conclusions are appropriately supported by the data. Since it uses a large cohort of patients, carries out a systematic evaluation across genetically defined categories, and critically evaluates diagnostic sensitivity and specificity this work has high translational value.
The authors inclused 204 fibroblast lines from genetically confirmed PMD patients, together with disease controls and healthy controls. The authors stratified the cohort into genetic and functional categories, including isolated respiratory chain defects, mitochondrial translation defects, mtDNA maintenance disorders, mtDNA variants, and other nuclear-encoded defects. This allowed the authors to provide useful conclusions regarding which classes of disorders are most reliably detected using fibroblast assays. The demonstration that combined functional testing achieves substantially higher diagnostic sensitivity than individual assays alone is particularly valuable and supports a multimodal diagnostic strategy.
Importantly, the authors used empirically derived thresholds to establish sensitivity and specificity in defining abnormal results. The authors also appropriately acknowledge the known challenges associated with fibroblast models, including tissue-specific expression differences and the instability of mtDNA heteroplasmy in cultured cells.
A challenge in current diagnostics is that genomic sequencing frequently identifies variants of uncertain significance or candidate disease genes that require functional validation. This study provides solid evidence of the reliability of fibroblast assays to give functional validation of VUS. The findings support the usefulness of the proposed pipeline to identify mitochondrial translation defects and isolated respiratory chain deficiencies, whereas mtDNA maintenance disorders are less reliably detected.
Despite these strengths, some relevant limitations should be addressed to further strengthen the manuscript. My main concern is that the prognostic value of the approach is not investigated. As all cases were genetically confirmed prior to functional analysis, the predictive value of the approach is not established. Functional testing in undiagnosed patients or patients with VUS

would increase the value of this work.
A second major concern is the reproducibility of the findings among laboratories. In 2013, Rodenburg and colleagues compared functional assays among several labs, highlighting large variability, which could be addressed by using normalization against a control sample. Since fibroblast functional assays are often performed in specialized laboratories. Discussing the potential variability between centers and the feasibility of standardization would enhance the clinical impact of the findings.

We thank the reviewers and the editor for their comments and the editor for further clarification. We address the comments point by point below. We provide a manuscript with the changes highlighted in yellow (except for the renumbering of the references as a result of inclusion of new references, and as a result of the reorganization of the structure of the manuscript with the methods placed after the discussion).

Referee #1 (Remarks for Author):
Question 0. The Authors demonstrate that a battery of assays, either separately or combined, can provide mostly good to excellent sensitivity to diagnose primary mitochondrial of multiple origins. The idea would before these assays, separately or combined, to be deployed when whole exome sequencing yields a novel variant in a nuclear DNA or mtDNA gene encoding a mitochondrial protein. This part of the study appears technically very well done, and the results are clear (though a little tedious to read, but the tabled information is clear), and potentially useful.
Answer 0. We thank the reviewer for this comment.

Question 1. Comments:1. A major concern is that many/most of these assays are not easy to perform by non-experts (including experience researchers in the mitochondrial biology field who do not have experience with these particular approaches) in a sufficiently optimized way as to minimize false negatives. EAs another example, BN-PAGE detection of assembled ETC complexes can require substantial optimization. Even if the methods were described in great detail (which they are not; only references are given), optimization of the aforementioned techniques would be needed. At the very least, the Authors should supply - possibly in a table - expected normal values (or a range) where possible, e.g. for enzyme activities.
Answer 1. In the Supplemental Information file, we had provided very detailed methods for each of the assays involved covering 16 pages long! We respectfully disagree that most methods are very tedious. The assays for the respiratory chain enzyme assays are performed in almost 20 clinical laboratories worldwide. The methods were originally obtained from the laboratory of Professor Thorburn with very minimal changes (for complex II, we use 100 μM coenzyme Q1 instead of 50 μM), and are similar to those performed in Newcastle and in Nijmegen. Professors Thorburn and Taylor have previously published these methods in great detail in (1) Frazier AE et al. Assessment of

mitochondrial respiratory chain enzymes in cells and tissues Method Cell Biol 2020;155:121-156, PMID 32183956 and in (2) Frazier AE, Thorburn Dr. Biochemical analyses of the electron transport chain complexes by spectrophotometry Methods Mol Biol 2012;837:49-62 PMID 22215540. As requested by the reviewer, we have added a table of normal values for our laboratory to this part of the method. One item to take into account is that the value for the combined complexes II-III assay depends to some extent on the fetal bovine serum equivalent used and its endogenous coenzyme Q content. When the supplier changed of our FBS, we had to make small changes to our normal values range for this assay. The pyruvate dehydrogenase enzyme assay is unchanged from that originally described by Professor Gary Brown and copied in most laboratories worldwide.

For the blue native PAGE with in-gel activity staining, the method was also previously described in great detail by group of Professor Van Coster in Smet, J., Devreese, B., and Van Coster, R. (2005) Nondenaturing polyacrylamide gel electrophoresis as a method for studying protein interactions: applications in the analysis of mitochondrial OXPHOS complexes. In: Celis, J.E. (ed) Cell Biology: a laboratory handbook, Academic, San Diego, pp 259-264 (reference 21). It is also described in great detail by us in the methods section. A normal result for this qualitative assay is already shown in Figure 2. We have found that this assay is actually easier to perform than the frequently published blue native PAGE followed by western blotting, which introduces considerably more variability in its transfer and detection system. Despite it having been reported since 2001 (reference 20), we find that this method has been somewhat underutilized in clinical laboratories, even though we find it easier to standardize than the commonly reported western blotting methods. Similarly, we have provided examples of normal results for the complex I assembly assay in Figure 2.

What is essential for all methods is to have sufficient starting material (outlined in the Tissue culture section in Methods). This is greatly facilitated by using a roller bottle system, an old technique (see for instance R.I. Fresney Animal Cell Culture, A practical approach, IRL Press, Oxford, 1986, pp.48-49). One T150 can be subcultured and expanded into one 480 cm^2 roller bottle and from there in one 850 cm^2 roller bottle. These roller bottles are flushed with a 5% CO2 mixture and tightly capped requiring only a heated chamber. This greatly decreases space in our tissue culture incubator, saves on technician time and tissue culture medium, and has greatly facilitated these assays. This has been described in the Tissue Culture section of our method description, and we have shared this information with several laboratories.

In contrast to the above assays, which were fairly easy to set up, we found two assays tedious: the complex V assay and the aconitase enzyme assay. We have described the optimization of our complex V method in detail in our previous publication including all normal values (reference 12). We found that using fresh material is important to have robust high values. Of note, this assay is in routine use in several European laboratories. The most difficult assay in our hands is the aconitase enzyme assay. We had already provided our current assay in the Methods section, and we have now added normal values for this assay. This assay is infrequently used.

<u>Question 2.</u> 2. Continuing with the previous comment, Authors should provide references that contain what Authors' judge to be "excellent" normal values from labs

that are deemed to run the tests well ("expert labs"). A list of such "expert" references can accompany each method, e.g., in a table. I think that this would be a relatively easy way to improve the utility of this study.

Answer 2. We find this a highly unusual request. It is not usual to provide the results from other laboratories that are not participants of this study. Moreover, small change in methods can provide for notable differences in normal values. Our method was derived from that of Professor Thorburn, and our values are within the same range, except for complex II where the increased amount of coenzyme Q1 in the assay in Colorado resulted in higher activity than the same assay in Melbourne. For comparison, we provide here the range provided by Prof Thorburn to us in 1997 (Thorbun DR, personal communication, with permission). Recent normal values from this laboratory can be found in PMID 36896486. We do not think though that it would be ethical to publish data from another laboratory that is not a participant in this manuscript.

	Colorado laboratory		Melbourne laboratory	
Complex	Avg	Range (P5-P95)	Avg	Range
Complex I	89.5	49.3-131.1	73.9	34-141
Complex II	234.0	130.9-364.4	55.5	22-100
Complex III	15.3	8.0-29.2	12.3	4.9-28.4
Complex II-III	107.8	61.8-158.8	71.4	40-122
Complex IV	4.6	2.2-7.1	5.72	1.10-11.6
Citrate synthase	407.5	253.5-554.1	211	87-322

Question 3. 3. The Abstract should state that analyses were performed in skin fibroblasts

Answer 3. We have now also added in the abstract that the analyses were performed in fibroblasts to the methods, in addition to the already mention in the conclusion. We had to move some words around to stay within the 175 word limit.

Question 4. 4. Should there be a recommendation as to the number of control samples to run for quantitative assays (e.g., enzyme activities)?

Answer 4. All assays include at least one normal control sample in each experiment for the respiratory chain enzyme assays, and this control should remain within the established normal range. For the complex V assay and for the PDH assay, each experiment includes both a normal control and a known deficient cell line. For the western blots, at least three normal controls are included in each experiment. This has been added to the methods description in the Open View. For the blue native PAGE with in-gel activity staining, one normal control is always included in each experiment. For the complex I assembly assay, all experiments include at least one normal control and one cell line cultured in the presence of a mitochondrial protein synthesis inhibitor (most commonly chloramphenicol) to visualize the abnormal bands. These methodological details were already listed in the Method description.

Question 5. 5. Should there be guidelines about culture conditions, especially substrate amounts (e.g., glucose concentration, inclusion or not of pyruvate) and confluence at the time of cell harvest?

Answer 5. These data were already provided in the Methods description in the section on Tissue culture.

Question 6. 6. In a few sentences, some rewording would improve clarity (e.g., in Discussion, para starting with "This study has two biases" - the second sentence (Cell lines with variants ...) could use some rewording).
Answer 6. Thank you for pointing out this issue. This sentence has been reworded. We hope that the intended message is now better understandable.

Referee #2 (Remarks for Author):
Question 0. This manuscript by Van Hove et al presents a broad-based biochemical approach to diagnosis of primary mitochondrial disease, which remains a significant challenge. The authors combine a variety of well-established biochemical approaches to present a compelling case for their proposed approach. The work captures the nuances involved in the diagnosis of a broad spectrum of disease, ranging from respiratory chain enzyme defects to mtDNA maintenance disorders. A particular strength of the work is the use of >300 fibroblast cell lines, with multiple lines for the different subsets of mitochondrial diseases. The development of such a testing regimen is important for labs tasked with identifying and diagnosing mitochondrial diseases of unknown etiology. While the analyses presented are all standard assays that have "stood the test of time", thus being robust and rigorous enough to function in the diagnostic process, the data support the combined approach in primary mitochondrial disease diagnosis. This work is likely to be of great interest for clinicians and clinical diagnostic labs focusing on mitochondrial disease.
Minor comments: The manuscript is clearly written and the conclusions drawn are supported by the data presented. There are, however, a couple of minor points that should be addressed:
Answer 0: We thank the reviewer for this summary.

Question 1: 1) Addressing some minor grammatical issues would improve manuscript flow i.e. abstract missing a couple of crucial commas.
Answer 1: The reviewer has not identified where a comma is missing. We have added a comma in the first sentence separating the two ideas: (1) variants of unknown significance require functional assays , and (2) genome sequencing does not identify variants in all patients. We have added a comma in the second sentence to separate the 204 PMD patients (which are divided into functional classes) from the controls and from the differential diagnostic conditions. If there are other places where a comma is needed, then we will gladly incorporate this.

Question 2: 2) It is disappointing that, in spite of the very large bibliography, there are no references to the seminal work done on mitochondrial disease diagnostics by the labs of Brian Robinson, Eric Shoubridge or Vamsi Mootha.
Answer: 2: The bibliography is not intended to be a historical review or to be comprehensive. Rather, it is focused specifically on those studies that provide systematic studies of the clinical utility of specific diagnostic modalities. The pioneering work in the next generation sequencing as a diagnostic tool for primary mitochondrial

disease by Professor Mootha was indeed cited in reference 24. The diagnostic pioneering of Professor Robinson for respiratory chain enzyme defects in fibroblasts is added as reference number 32. Whereas Professor Shoubridge has published very extensively about various genetic causes including using fibroblasts studies, we could not identify any publication that addresses sensitivity or specificity of fibroblast functional assays to diagnose primary mitochondrial diseases, that would be applicable for this manuscript. If such a manuscript could be identified, we will gladly cite it.

Question 3: 3) The use of antibiotics in the fibroblast cultures needs further explanation, given that some antibiotics have been shown to negatively affect mitochondrial function.
Answer 3: Indeed it is known that streptomycin may at high concentrations inhibits mitochondrial ribosomal function. First, all controls and all patients' cells have always been cultured in an identical way such that if there is a depressing activity, it would be equally applicable to both patients and controls. Second, to evaluate the impact of antibiotics and antimycotics, five different cell lines were cultured for three days either with antibiotics and antimycotics and in parallel without antibiotics and antimycotics. The respiratory chain enzyme activities were measured and compared using a paired t-test. No significant differences were identified (all p-values >0.14). Of note, in the interlaboratory comparison by Rodenburg et al (reference 85), all laboratories use the same antibiotics and antimycotics in the culture of their fibroblasts.

Complex	without AB/AM*	with AB/AM*	p-value#
Complex I	147±54	135±41	0.78 / 0.69
Complex II	252±84	247±31	0.84 / 0.89
Complex III	16.1±5.1	13.7±2.6	0.17 / 0.22
Complex II-III	132±39	127±32	0.51 / 0.50
Complex IV	5.58±1.39	4.78±1.00	0.59/ 0.14
Citrate synthase	514±156	470±58	0.71 / 0.50

Abbreviations: * AB/AM is antibiotics and antimycotics, # p-values given for paired Student t-test / related samples Wilcoxon signed rank test.

Question 4: 4) Cutoffs optimization: "...a gradual increase for complex IV....with no impact on complexes II and IV..." Did you mean Complex V?
Answer 4: Thank you for observing this error. This should be "…with no impact on complex II." We indeed already described complex IV before. The cutoff for complex V is a separate issue, extensively discussed in the manuscript reference number 39. There the cutoff is based on the difference between controls which ranges from 65% to 140% of the average of controls, and is handled separately from the other complexes and is not intended in the analysis here. This has been corrected in the manuscript.

Referee #3 (Comments on Novelty/Model System for Author):
Question 0: The methodologies used are well established and solid. The study is not new, as fibroblasts and used assays have been used for decades, but the study may provide a general frame for the diagnosis of PMDs.
Referee #3 (Remarks for Author):The manuscript by Van Hove and colleagues presents a comprehensive evaluation of functional mitochondrial assays performed in fibroblasts derived from a large cohort of patients with genetically confirmed primary mitochondrial

disease (PMD), alongside disease controls and healthy controls. Fibroblasts from patients have been used as disease models for decades and have been an extremely useful tool to study the consequences of mutations. The authors systematically assess the diagnostic performance of respiratory chain enzyme activity measurements, blue native PAGE in-gel activity assays, complex I assembly analysis, and targeted protein abundance quantification. Overall, although not novel as methodology and outcome, the study addresses the very relevant diagnostic utility of functional validation in fibroblasts in the era of next-generation sequencing. The is provides important quantitative evidence supporting the continued role of fibroblast-based functional assays in the diagnostic evaluation of mitochondrial disease. The manuscript is clearly written, the experimental approach is methodologically sound, and the conclusions are appropriately supported by the data. Since it uses a large cohort of patients, carries out a systematic evaluation across genetically defined categories, and critically evaluates diagnostic sensitivity and specificity this work has high translational value. The authors included 204 fibroblast lines from genetically confirmed PMD patients, together with disease controls and healthy controls. The authors stratified the cohort into genetic and functional categories, including isolated respiratory chain defects, mitochondrial translation defects, mtDNA maintenance disorders, mtDNA variants, and other nuclear-encoded defects. This allowed the authors to provide useful conclusions regarding which classes of disorders are most reliably detected using fibroblast assays. The demonstration that combined functional testing achieves substantially higher diagnostic sensitivity than individual assays alone is particularly valuable and supports a multimodal diagnostic strategy. Importantly, the authors used empirically derived thresholds to establish sensitivity and specificity in defining abnormal results. The authors also appropriately acknowledge the known challenges associated with fibroblast models, including tissue-specific expression differences and the instability of mtDNA heteroplasmy in cultured cells. A challenge in current diagnostics is that genomic sequencing frequently identifies variants of uncertain significance or candidate disease genes that require functional validation. This study provides solid evidence of the reliability of fibroblast assays to give functional validation of VUS. The findings support the usefulness of the proposed pipeline to identify mitochondrial translation defects and isolated respiratory chain deficiencies, whereas mtDNA maintenance disorders are less reliably detected.
<u>Answer 0:</u> Thank you for this summary of our manuscript.

<u>Question 1:</u> Despite these strengths, some relevant limitations should be addressed to further strengthen the manuscript. My main concern is that the prognostic value of the approach is not investigated. As all cases were genetically confirmed prior to functional analysis, the predictive value of the approach is not established. Functional testing in undiagnosed patients or patients with VUS would increase the value of this work.
<u>Answer 1:</u> We agree that a prospective study of blinded cases where the genetics is not yet known at the time of the analysis followed by genetic studies would be useful in order to determine the predictive value of an abnormal result. We have added this as a limitation of our study. We would point out that this study would be difficult to organize in our country where genetic testing precedes any functional testing as outlined in the

paradigm, and hence biopsied materials for fibroblasts are generally not available before genetic testing.
This has been added to the discussion as follows: “A limitation of our study is that it is retrospective and used samples where the genetics was already known. A prospective study would be useful to determine the predictive value of an abnormal result.”

Question 2: A second major concern is the reproducibility of the findings among laboratories. In 2013, Rodenburg and colleagues compared functional assays among several labs, highlighting large variability, which could be addressed by using normalization against a control sample. Since fibroblast functional assays are often performed in specialized laboratories. Discussing the potential variability between centers and the feasibility of standardization would enhance the clinical impact of the findings
Answer 2: Indeed, there are differences between methods used in various laboratories that result in different normal and pathological values. Given the very extensive requirements for validation of these tests, it is unlikely that laboratories will change the small methodological changes as outlined by Rodenburg et al. Whereas the buffer system has little influence on the activities, differences in mitochondrial isolation and in the specific assays influence the specific activities reported. Increasingly, laboratories are using direct assays for complex I and for complex III, rather than using combined linked assays (NADH:cytochrome c oxidoreductase (I-III combined assay) and succinate:cytochrome c oxidoreductase (II-III). As published before, combined assays have less sensitivity to detect deficiencies, and in our study too, isolated complex III deficiency was systematically missed using the combined II-III assay whereas it was mostly identified with the isolated complex III assay. This has been attributed to the more stringent control that complex II has over the combined assay than complex III (ref. 89).
Provided that laboratories use these isolated assays, despite the differences in absolute activities, the relative values and particularly the internal ratios over citrate synthase appeared to be similar across the different laboratories, and the laboratories equally detected deficiencies correctly. In our experience of where we have actively collaborated with other laboratories, provided the laboratories use isolated complex assays, the performance is not different. The controls remain controls, affected patients exhibit the same deficiencies, and borderline results remain borderline. Even though the actual numbers are different, the distinction between controls and affected patients are surprisingly similar. This experience is indeed what Rodenburg et al published in 2013. All clinical laboratories perform QC schematics, but an interlaboratory QC scheme is not available. Of note, the Rodenburg et al publication only recommends to include a normal control if laboratories want to compare direct values of a specific patient between the two laboratories, but does not suggest that this should be a routine part of clinical procedures. As noted before in Reviewer 1 question 4, all our assays include at least a normal control as part of our standard QC. This has been summarized and added to the discussion as follows:
“Interlaboratory comparisons have highlighted differences in methodology between clinical laboratories performing these mitochondrial functional assays[86,87]. In these studies, recognition of complex I and complex III assays was superior in direct isolated

assays than in combined linked I-III or II-III assays[87,88], which we also observed. For complex III[88,89] , this relates in part to locus of control[90]. Provided laboratories use similar isolated complex I and complex III assays, in interlaboratory comparative studies, different laboratories made similar diagnostic discrimination between controls and affected patients despite these small technical differences[86.87], which were mostly attributed to differences in mitochondrial isolation[86]. The current study documents the clinical validity of such functional assays."

Editor comments:
We require:
1) A .docx formatted version of the manuscript text (including legends for main figures, EV figures and tables). Please make sure that the changes are highlighted to be clearly visible.
Answer 1: The manuscript text is provided as a .docx. All figures with legends and tables with legends are included. Any changes to the text are highlighted in yellow.
The manuscript is provided in a .docx file. Following discussion with the editor, we have moved all methodological details from Expanded view and incorporated this information into the Methods section of the manuscript. In keeping with the request by the reviewers and to enable replication, the methods were provided in sufficient detail. This has obviously expanded the word count. In keeping with the style of the journal, we have moved the methods section to after the discussion. In order to keep full understanding, a small part about the setup of the study that was present in the Methods section is now added to the end of the introduction, and we also reviewed that all abbreviations are spelled out at first encounter, and not only in the methods section. The Table of all Reagents and a Tables of Antibodies used is listed as an Excel file in Appendix Tables. Per discussion with the editor, we have moved the four Expanded View tables into an Excel format and added the legend into the Excel spreadsheet. The other tables that were part of Supplemental Materials have been moved to Appendix tables. As discussed with the editor, the Strobe checklist has also been moved to Appendix file. We have changed the reference style in both the manuscript text and in the references to accommodate the journal style.

2) Individual production quality figure files as .eps, .tif, .jpg (one file per figure). For guidance, download the 'Figure Guide PDF':
https://media.springernature.com/original/springer-cms/rest/v1/content/27825798/data/v1.
Answer 2: All figures are provided as high quality .tif files.
For figure 1, we have removed the gels and blots, since this is a schematic and these gels/blots are part of figure 2.

3) A .docx formatted letter INCLUDING the reviewers' reports and your detailed point-by-point responses to their comments. As part of the EMBO Press transparent editorial process, the point-by-point response is part of the Review Process File (RPF), which will be published alongside your paper.
Answer 3: We have provided a detailed point-by-point response to the questions raised by the reviewers.

4) A complete author checklist, which you can download from our author guidelines. Please insert information in the checklist that is also reflected in the manuscript. The completed author checklist will also be part of the RPF.
Answer 4: A complete author checklist is provided. A statement on sample size and on blinding is added per the checklist.

5) Please note that all corresponding authors are required to supply an ORCID ID for their name upon submission of a revised manuscript.
Answer 5: A complete list of each author's ORCID ID is provided at the end of this letter.

6) It is mandatory to include a 'Data Availability' section after the Materials and Methods. Before submitting your revision, primary datasets produced in this study need to be deposited in an appropriate public database, and the accession numbers and database listed under 'Data Availability'. Please remember to provide a reviewer password if the datasets are not yet public. In case you have no data that requires deposition in a public database, please state so in this section. Note that the Data Availability Section is restricted to new primary data that are part of this study.
Answer 6: All pertinent primary data are included in EV tables 2 and 3. In addition, results of most blots are deposited in Zenodo at 10.5281/zenodo.20615940. The data can be reviewed at
https://zenodo.org/records/20615940?preview=1&token=eyJhbGciOiJIUzUxMiJ9.eyJpZCI6ImRjNTdkNDE3LTNiNGEtNGI3YS1hYWI0LWU0ZjhiZjdiNzZjYiIsImRhdGEiOnt9LCJyYW5kb20iOiI5ZmVjOGE3NjhjNTI0NDA0NzNiZTQ2ZGFhMWRjNTgyOSJ9.c3qdC7fXuOPUFhOwZuO14Yq5ncFv9qjm9Nh-Q1caNpnF94dFQSwPejHj9U7zk0Cnj20CgS3ixuwt07hcjIxv4A.
Otherwise, this repository is restricted for access until the manuscript is published, and we will make it available at that time. It will be important for the publisher to timely let us know which date this will be so that we can coordinate for the release of the data.

7) For data quantification: please specify the name of the statistical test used to generate error bars and P values, the number (n) of independent experiments (specify technical or biological replicates) underlying each data point and the test used to calculate p-values in each figure legend. The figure legends should contain a basic description of n, P and the test applied. Graphs must include a description of the bars and the error bars (s.d., s.e.m.).
Answer 7: All statistical analysis is limited to descriptive information. At each point, the number of data is provided (when added, highlighted in yellow).

8) At EMBO Press we ask authors to provide source data for the main manuscript figures. You will receive a separate email with instructions for providing source data with your revised manuscript, including how to upload and organize the files.
Answer 8: A zip file including the source data of the main manuscript figure 2 is provided. Figure 1 is now exclusively a schematic without source data.

9) Our journal encourages inclusion of *data citations in the reference list* to directly cite datasets that were re-used and obtained from public databases. Data citations in the article text are distinct from normal bibliographical citations and should directly link to the database records from which the data can be accessed. In the main text, data citations are formatted as follows: "Data ref: Smith et al, 2001"or "Data ref: NCBI Sequence Read Archive PRJNA342805, 2017". In the Reference list, data citations must be labeled with "[DATASET]". A data reference must provide the database name, accession number/identifiers and a resolvable link to the landing page from which the data can be accessed at the end of the reference.
Answer 9: Not applicable. No public dataset was re-used.

10) We replaced Supplementary Information with Expanded View (EV) Figures and Tables that are collapsible/expandable online. A maximum of 5 EV Figures can be typeset. EV Figures should be cited as 'Figure EV1, Figure EV2" etc... in the text and their respective legends should be included in the main text after the legends of regular figures.- For the figures that you do NOT wish to display as Expanded View figures, they should be bundled together with their legends in a single PDF file called *Appendix*, which should start with a short Table of Content. Appendix figures should be referred to in the main text as: "Appendix Figure S1, Appendix Figure S2" etc.- Additional Tables/Datasets should be labeled and referred to as Table EV1, Dataset EV1, etc. Legends have to be provided in a separate tab in case of .xls files. Alternatively, the legend can be supplied as a separate text file (README) and zipped together with the Table/Dataset file.
Answer 10: There are no EV Figures. There are 4 tables provided as EV labeled in the text as Tables EV1 through Table EV4. These four tables are provided as Excel files .xls. Legends are provided in a separate tab.
We have moved the remaining two tables to Appendix tables 2 and 3.

11) The paper explained: EMBO Molecular Medicine articles are accompanied by a summary of the articles to emphasize the major findings in the paper and their medical implications for the non-specialist reader. Please provide a draft summary of your article highlighting- the medical issue you are addressing,- the results obtained and- their clinical impact. This may be edited to ensure that readers understand the significance and context of the research. Please refer to any of our published articles for an example.
Answer 11: A Paper Explained has been provided.

12) Author contributions: You will be asked to provide CRediT (Contributor Role Taxonomy) terms in the submission system. These replace a narrative author contribution section in the manuscript.
Answer: 12: We will provide this in the system. We have left the current description in case any clarity if needed. It can be removed by the publisher as needed.

13) A Conflict of Interest statement should be provided in the main text.
Answer 12: A conflict-of-interest statement as it pertains to this study is provided in the main text immediately after Acknowledgment. No author had a conflict of interest as it

pertains to this specific study. After discussion with the Editor, any further notes were removed.

14) Every published paper includes a 'Synopsis' to further enhance discoverability. Synopses are displayed on the journal webpage and are freely accessible to all readers. They include a short stand first (maximum of 300 characters, including space) as well as 2-5 one-sentences bullet points that summarizes the paper. Please write the bullet points to summarize the key NEW findings. They should be designed to be complementary to the abstract - i.e. not repeat the same text. We encourage inclusion of key acronyms and quantitative information (maximum of 30 words / bullet point). Please use the passive voice. Please attach these in a separate file or send them by email, we will incorporate them accordingly. Please also suggest a striking image or visual abstract to illustrate your article as a PNG file 550 px wide x 300-600 px high.
Answer 14:
A synopsis is provided.
As discussed with the editor, we propose to use Figure 1 as the visual abstract since it reflects and summarizes the study results and its context well, but maintains the figures of blots from figure 2. We have exported this into a .png file at high resolution. I am not aware how to have it a specific pixel dimension.

15) Include a Reagents and Tools Table as part of the Methods section, which can be downloaded from our author guidelines.
Answer 15: Given the large number of reagents, these tables are provided in the Appendix Table 1. The first tab provides the reagents, the second tab provides the antibodies used.

We hope that these revisions adequately address the concerns raised by the reviewers and that they will make the manuscript acceptable for publication. Feel free to contact us should there be any further questions.

7th Jul 2026

Dear Dr. Van Hove,

Thank you for the submission of your revised manuscript to EMBO Molecular Medicine. I am pleased to inform you that we will be able to accept your manuscript pending the following final amendments:

1) Please address referee #1 minor points.
2) Authors: E-mail correspondence to Kristen Wigby could not be delivered. Please update the e-mail address and make sure to enter correct e-mail addresses for all authors in our submission system.
3) Please consider revising the title to better highlight the main findings, e.g. "Comprehensive functional testing in fibroblasts improves the diagnosis of primary mitochondrial disease".
4) Abstract: I have gone through your text and revised it (see below). Please review it and amend as you see fit:

Genome sequencing has transformed the diagnosis of primary mitochondrial diseases (PMDs), yet many patients remain without a molecular diagnosis because of variants of uncertain significance or unresolved genetic findings. Here, we systematically evaluated the clinical performance of a comprehensive fibroblast-based functional testing pipeline in a cohort of 204 patients with genetically confirmed PMD, 51 healthy controls, and 53 patients with differential diagnoses. The testing battery comprised respiratory chain enzyme assays, blue native polyacrylamide gel electrophoresis with in-gel activity staining, complex I assembly analysis, and targeted assessment of mitochondrial protein abundance. Individually, respiratory chain enzyme assays, BN-PAGE, and complex I assembly analysis showed sensitivities of 46%, 40%, and 49%, respectively, with specificities of 93-99%. Combined, the assays achieved an overall sensitivity of 76%, specificity of 93%, positive predictive value of 96%, and negative predictive value of 67%. Diagnostic performance was highest for isolated respiratory chain deficiencies, nuclear DNA-encoded mitochondrial translation defects, cofactor deficiencies, and mitochondrial aminoacyl-tRNA synthetase disorders, whereas mitochondrial DNA variants and maintenance defects remained challenging. Secondary mitochondrial dysfunction were rare. These findings establish comprehensive fibroblast functional testing as a robust complement to genomic sequencing, enabling clinically meaningful interpretation of genetic findings and improving the diagnosis of primary mitochondrial disease.

5) In the main manuscript file, please do the following:
- Remove One Sentence Summary from the manuscript title page.
- Remove the list of abbreviations and incorporate it into the text.
- In Methods, provide the statement that the experiments involving human subjects conformed to the principles set out in the WMA Declaration of Helsinki and the Department of Health and Human Services Belmont Report.
- Indicate in legends number and nature of replicates and exact p= values, not a range, along with the statistical test used. To keep the figures "clear" some authors found providing an Appendix table Sx with all exact p-values preferable. You are welcome to do this if you want to.
- Author contributions: Please remove it from the manuscript and specify author contributions in our submission system. CRediT has replaced the traditional author contributions section because it offers a systematic machine-readable author contributions format that allows for more effective research assessment. You are encouraged to use the free text boxes beneath each contributing author's name to add specific details on the author's contribution. More information is available in our guide to authors:
https://www.embopress.org/page/journal/17574684/authorguide#authorshipguidelines
- Please make sure that all deposited data are freely accessible.
6) Reagent table: Reagents Table will be typeset and follows a standardized format, therefore please transfer the information from this table to our reagents and tools table template. Please remove appendix table 1 from the file name. More information on how to adhere to this format as well as downloadable templates (.docx) for the Reagents and Tools Table can be found in our author guidelines:
https://www.embopress.org/page/journal/17574684/authorguide#structuredmethods
7) Tables:
- Please rename Tables EV1 - EV3 and upload them as Dataset EV1 - Dataset EV3. Please correct the nomenclature in the files' legends, as well as the callouts in the manuscript text. Please rename the last expanded view table to Table EV1.
- Please upload Appendix Table 3 and 4 as separate files and rename them to Table EV2 and Table EV3. Please update their callouts in the main text.
- Please remove appendix STROBE checklist.
8) The paper Explained: Please add it to the main manuscript file.
9) Synopsis:
- Synopsis image: Please resize the image to exactly 550 px-wide x 300 pixels high and upload it as a high-resolution .jpeg/.png file. Please note that the image may need to be simplified in order to remain legible in this small size.
- Please check your synopsis text and image before submission with your revised manuscript. Please be aware that in the proof stage minor corrections only are allowed (e.g., typos).
10) As part of the EMBO Publications transparent editorial process (see our Editorial at http://embomolmed.embopress.org/content/2/9/329), EMBO Molecular Medicine will publish online a Review Process File (RPF)

to accompany accepted manuscripts. This file will be published in conjunction with your paper and will include the anonymous referee reports, your point-by-point response and all pertinent correspondence relating to the manuscript. Let us know if you want to remove or not any figures from it prior to publication. Please note that the Authors checklist will be published at the end of the RPF.
11) Please provide a point-by-point letter INCLUDING my comments as well as the reviewer's reports and your detailed responses (as Word file).

I look forward to reading a new revised version of your manuscript as soon as possible.

Yours sincerely,

Zeljko Durdevic

Zeljko Durdevic
Senior Editor
EMBO Molecular Medicine

*** Instructions to submit your revised manuscript ***

*** PLEASE NOTE *** As part of the EMBO Publications transparent editorial process initiative (see our Editorial at https://www.embopress.org/doi/pdf/10.1002/emmm.201000094), EMBO Molecular Medicine will publish online a Review Process File to accompany accepted manuscripts.

In the event of acceptance, this file will be published in conjunction with your paper and will include the anonymous referee reports, your point-by-point response and all pertinent correspondence relating to the manuscript. If you do NOT want this file to be published, please inform the editorial office at contact@embomolmed.org.

*****

When preparing your revised manuscript, please refer to our guidelines: https://link.springer.com/journal/44321/submission-guidelines#cms-Revised-submissions. We perform an initial quality control of all revised manuscripts before re-review; failure to include requested items will delay the evaluation of your revision.

We require:

1) A .docx formatted version of the manuscript text (including legends for main figures, EV figures and tables). Please make sure that the changes are highlighted to be clearly visible.

2) Individual production quality figure files as .eps, .tif, .jpg (one file per figure). For guidance, download the 'Figure Guide PDF': https://media.springernature.com/original/springer-cms/rest/v1/content/27825798/data/v1.

3) A .docx formatted letter INCLUDING the reviewers' reports and your detailed point-by-point responses to their comments. As part of the EMBO Press transparent editorial process, the point-by-point response is part of the Review Process File (RPF), which will be published alongside your paper.

4) A complete author checklist, which you can download from our author guidelines. Please insert information in the checklist that is also reflected in the manuscript. The completed author checklist will also be part of the RPF.

5) Please note that all corresponding authors are required to supply an ORCID ID for their name upon submission of a revised manuscript.

6) It is mandatory to include a 'Data Availability' section after the Materials and Methods. Before submitting your revision, primary datasets produced in this study need to be deposited in an appropriate public database, and the accession numbers and database listed under 'Data Availability'. Please remember to provide a reviewer password if the datasets are not yet public.

In case you have no data that requires deposition in a public database, please state so in this section. Note that the Data Availability Section is restricted to new primary data that are part of this study.

7) For data quantification: please specify the name of the statistical test used to generate error bars and P values, the number (n) of independent experiments (specify technical or biological replicates) underlying each data point and the test used to calculate p-values in each figure legend. The figure legends should contain a basic description of n, P and the test applied. Graphs must include a description of the bars and the error bars (s.d., s.e.m.).

8) At EMBO Press we ask authors to provide source data for the main manuscript figures. You will receive a separate email with instructions for providing source data with your revised manuscript, including how to upload and organize the files.

9) Our journal encourages inclusion of *data citations in the reference list* to directly cite datasets that were re-used and obtained from public databases. Data citations in the article text are distinct from normal bibliographical citations and should directly link to the database records from which the data can be accessed. In the main text, data citations are formatted as follows: "Data ref: Smith et al, 2001" or "Data ref: NCBI Sequence Read Archive PRJNA342805, 2017". In the Reference list, data citations must be labeled with "[DATASET]". A data reference must provide the database name, accession number/identifiers and a resolvable link to the landing page from which the data can be accessed at the end of the reference.

10) We replaced Supplementary Information with Expanded View (EV) Figures and Tables that are collapsible/expandable online. A maximum of 5 EV Figures can be typeset. EV Figures should be cited as 'Figure EV1, Figure EV2" etc... in the text and their respective legends should be included in the main text after the legends of regular figures.

- For the figures that you do NOT wish to display as Expanded View figures, they should be bundled together with their legends in a single PDF file called *Appendix*, which should start with a short Table of Content. Appendix figures should be referred to in the main text as: "Appendix Figure S1, Appendix Figure S2" etc.

- Additional Tables/Datasets should be labeled and referred to as Table EV1, Dataset EV1, etc. Legends have to be provided in a separate tab in case of .xls files. Alternatively, the legend can be supplied as a separate text file (README) and zipped together with the Table/Dataset file.

11) The paper explained: EMBO Molecular Medicine articles are accompanied by a summary of the articles to emphasize the major findings in the paper and their medical implications for the non-specialist reader. Please provide a draft summary of your article highlighting

- the medical issue you are addressing,

- the results obtained and

- their clinical impact.

This may be edited to ensure that readers understand the significance and context of the research. Please refer to any of our published articles for an example.

12) Author contributions: You will be asked to provide CRediT (Contributor Role Taxonomy) terms in the submission system. These replace a narrative author contribution section in the manuscript.

13) A Conflict of Interest statement should be provided in the main text.

14) Every published paper includes a 'Synopsis' to further enhance discoverability. Synopses are displayed on the journal webpage and are freely accessible to all readers. They include a short stand first (maximum of 300 characters, including space) as well as 2-5 one-sentences bullet points that summarizes the paper. Please write the bullet points to summarize the key NEW findings. They should be designed to be complementary to the abstract - i.e. not repeat the same text. We encourage inclusion of key acronyms and quantitative information (maximum of 30 words / bullet point). Please use the passive voice. Please attach these in a separate file or send them by email, we will incorporate them accordingly.

Please also suggest a striking image or visual abstract to illustrate your article as a PNG file 550 px wide x 300 px high.

15) Include a Reagents and Tools Table as part of the Methods section, which can be downloaded from our author guidelines.

EMBO Molecular Medicine has a "scooping protection" policy, whereby similar findings that are published by others during review or revision are not a criterion for rejection. Should you decide to submit a revised version, I do ask that you get in touch after three months if you have not completed it, to update us on the status.

*Additional important information regarding Figures

Each figure should be given in a separate file and should have the following resolution:
Graphs 800-1,200 DPI
Photos 400-800 DPI
Colour (only CMYK) 300-400 DPI"

Figures are not edited by the production team. All lettering should be the same size and style; figure panels should be indicated by capital letters (A, B, C etc). Gridlines are not allowed except for log plots. Figures should be numbered in the order of their appearance in the text with Arabic numerals. Each Figure must have a separate legend and a caption is needed for each panel.

*Additional important information regarding figures and illustrations can be found at
https://media.springernature.com/original/springer-cms/rest/v1/content/27825798/data/v1

***** Reviewer's comments *****

Referee #1 (Comments on Novelty/Model System for Author):

The reviewers have addressed all my concerns.

Referee #1 (Remarks for Author):

Thank you for addressing my comments.
Please, just a note about Question 2: My idea was to include published values from experienced labs; I don't see an ethical problem with that, as long as it is made clear that the data are published already.
Also, please note: Concerning my points 1 and 2, unfortunately I see a certain number of instances of people performing ETC complex activity experiments or O2 consumption experiments without checking their control values against the literature, and are satisfied to report that a certain model shows no change relative to a control that is already very low. This type of situation is the basis for my questions.

Referee #2 (Comments on Novelty/Model System for Author):

HIgh technical quality and rigorous analyses; authors have adequately addressed comments from first review.

Referee #2 (Remarks for Author):

Thank you for addressing my concerns and amending the manuscript accordingly.

1) Please address referee #1 minor points.

Thank you for addressing my comments. Please, just a note about Question 2: My idea was to include published values from experienced labs; I don't see an ethical problem with that, as long as it is made clear that the data are published already. Also, please note: Concerning my points 1 and 2, unfortunately I see a certain number of instances of people performing ETC complex activity experiments or O2 consumption experiments without checking their control values against the literature, and are satisfied to report that a certain model shows no change relative to a control that is already very low. This type of situation is the basis for my questions.

Answer:

We share the concerns of the reviewer. In addition, we add to this the concern of comparing to only a very limited number of controls, which often does not adequately reveal the span of the controls. For instance, we evaluated oxygen consumption rate using Oxygraph O2k with the commonly used SUIT protocol (see a.o. PMID 32931937 and PMID 30283131). When comparing an individual patient to a limited set of controls, this appears to work well. However, when we reviewed 41 controls, despite our best efforts at standardization, the normal range widens, and less than 20% of patients had abnormal values. There was no added benefit beyond the already established assays described here to identify additional not yet diagnosed patients. Revisiting electron transport chain enzyme assays, the normal values are highly dependent on details of the method used. As was cited in our previous answer, this is highly dependent on details of the sample preparation. Even small differences, in our case increasing coenzyme Q_1 substrate concentration in the complex II assay compared to the laboratory of Professor Thorburn, already substantially altered the normal range. It would not make sense in this publication to cite normal values of other methods used, since these would need to be matched to detailed descriptions of those other methods. Moreover, this is not the forum to solve this real issue, as we are not comparing other methods of enzyme assays here. For those willing to use our assays and methods, we have provided here in our methods section very detailed methodological descriptions, and, further, upon request, we have repeatedly shared the complete laboratory method in CAP style. This should easily enable anyone who wants to use our method to compare their results with our findings.

22nd Jul 2026

Dear Dr. Van Hove,

We are pleased to inform you that your manuscript is accepted for publication and is now being sent to our publisher to be included in the next available issue of EMBO Molecular Medicine.

Your manuscript will be processed for publication by EMBO Press. It will be copy edited and you will receive page proofs prior to publication. Please note that you will be contacted by Springer Nature Author Services to complete licensing and payment information.

You may qualify for financial assistance for your publication charges - either via a Springer Nature fully open access agreement or an EMBO initiative. Check your eligibility: https://link.springer.com/journal/44321/how-to-publish-with-us

Should you be planning a Press Release on your article, please notify the production team via this form in order to coordinate publication and release dates: https://forms.cloud.microsoft/e/eH4A8JVP6g

If you have any questions, please do not hesitate to contact the Editorial Office. Thank you for your contribution to EMBO Molecular Medicine.

Yours sincerely,
Zeljko Durdevic

Zeljko Durdevic
Senior Editor
EMBO Molecular Medicine

>>> Please note that it is EMBO Molecular Medicine policy for the transcript of the editorial process (containing referee reports and your response letter) to be published as an online supplement to each paper. If you do NOT want this, you will need to inform the Editorial Office via email immediately. More information is available here: https://link.springer.com/partners/embo-press/editorial-policies#Peer%20review

EMBO Press Author Checklist

Corresponding Author Name: Johan L.K. Van Hove	
Journal Submitted to: EMBO Molecular Medicine	
Manuscript Number: EMM-2026-23411	

USEFUL LINKS FOR COMPLETING THIS FORM
The EMBO Journal - Author Guidelines
EMBO Reports - Author Guidelines
Molecular Systems Biology - Author Guidelines
EMBO Molecular Medicine - Author Guidelines

Reporting Checklist for Life Science Articles (updated January
This checklist is adapted from Materials Design Analysis Reporting (MDAR) Checklist for Authors. MDAR establishes a minimum set of requirements in transparent reporting in the life sciences (see Statement of Task: 10.31222/osf.io/9sm4x). Please follow the journal's guidelines in preparing your
Please note that a copy of this checklist will be published alongside your article.

Abridged guidelines for figures
1. Data
The data shown in figures should satisfy the following conditions:
- the data were obtained and processed according to the field's best practice and are presented to reflect the results of the experiments in an accurate and unbiased manner.
- ideally, figure panels should include only measurements that are directly comparable to each other and obtained with the same assay.
- plots include clearly labeled error bars for independent experiments and sample sizes. Unless justified, error bars should not be shown for technical
- if n<5, the individual data points from each experiment should be plotted. Any statistical test employed should be justified.
- Source Data should be included to report the data underlying figures according to the guidelines set out in the authorship guidelines on Data

2. Captions
Each figure caption should contain the following information, for each panel where they are relevant:
- a specification of the experimental system investigated (eg cell line, species name).
- the assay(s) and method(s) used to carry out the reported observations and measurements.
- an explicit mention of the biological and chemical entity(ies) that are being measured.
- an explicit mention of the biological and chemical entity(ies) that are altered/varied/perturbed in a controlled manner.
- the exact sample size (n) for each experimental group/condition, given as a number, not a range;
- a description of the sample collection allowing the reader to understand whether the samples represent technical or biological replicates (including how many animals, litters, cultures, etc.).
- a statement of how many times the experiment shown was independently replicated in the laboratory.
- definitions of statistical methods and measures:
  - common tests, such as t-test (please specify whether paired vs. unpaired), simple $\chi 2$ tests, Wilcoxon and Mann-Whitney tests, can be unambiguously identified by name only, but more complex techniques should be described in the methods section;
  - are tests one-sided or two-sided?
  - are there adjustments for multiple comparisons?
  - exact statistical test results, e.g., P values = x but not P values < x;
  - definition of 'center values' as median or average;
  - definition of error bars as s.d. or s.e.m.

Please complete ALL of the questions below.
Select "Not Applicable" only when the requested information is not relevant for your study.

Materials

Newly Created Materials	Information included in the manuscript?	In which section is the information available? (Reagents and Tools Table, Materials and Methods, Figures, Data Availability Section)
New materials and reagents need to be available; do any restrictions apply?	Not Applicable	No new materials were generated or used.

Antibodies	Information included in the manuscript?	In which section is the information available? (Reagents and Tools Table, Materials and Methods, Figures, Data Availability Section)
For **antibodies** provide the following information:<br>- Commercial antibodies: RRID (if possible) or supplier name, catalogue number and or/clone number<br>- Non-commercial: RRID or citation	Yes	All reagents and all antibodies are listed in a Table in Appendix. For antibodies, the supplier, catalog number and dilution used are provided.

DNA and RNA sequences	Information included in the manuscript?	In which section is the information available? (Reagents and Tools Table, Materials and Methods, Figures, Data Availability Section)
**Short novel DNA or RNA including primers, probes**: provide the sequences.	Not Applicable	

Cell materials	Information included in the manuscript?	In which section is the information available? (Reagents and Tools Table, Materials and Methods, Figures, Data Availability Section)
**Cell lines:** Provide species information, strain. Provide accession number in repository **OR** supplier name, catalog number, clone number, and/**OR** RRID.	Not Applicable	No cell lines were obtained from a repository
**Primary cultures:** Provide species, strain, sex of origin, genetic modification status.	Yes	All cultures are of human fibroblasts. Sex and age in years as available is provided in Table EV1
Report if the cell lines were recently **authenticated** (e.g., by STR profiling) and tested for mycoplasma contamination.	Yes	All cell lines were routinely tested for mycoplasma. STR profiling was not done.

Experimental animals	Information included in the manuscript?	In which section is the information available? (Reagents and Tools Table, Materials and Methods, Figures, Data Availability Section)
**Laboratory animals or Model organisms:** Provide species, strain, sex, age, genetic modification status. Provide accession number in repository **OR** supplier name, catalog number, clone number, **OR** RRID.	Not Applicable	
**Animal observed in or captured from the field:** Provide species, sex, and age where possible.	Not Applicable	
Please detail **housing and husbandry conditions**.	Not Applicable	

Plants and microbes	Information included in the manuscript?	In which section is the information available? (Reagents and Tools Table, Materials and Methods, Figures, Data Availability Section)
**Plants:** provide species and strain, ecotype and cultivar where relevant, unique accession number if available, and source (including location for collected wild specimens).	Not Applicable	
**Microbes:** provide species and strain, unique accession number if available, and source.	Not Applicable	

Human research participants	Information included in the manuscript?	In which section is the information available? (Reagents and Tools Table, Materials and Methods, Figures, Data Availability Section)
If collected and within the bounds of privacy constraints report on age, sex and gender or ethnicity for all study participants.	Yes	This is provided in Table EV1. This includes age and sex. Race and Ethinicity was not collected since this was a secndarily deidentified dataset.

Core facilities	Information included in the manuscript?	In which section is the information available? (Reagents and Tools Table, Materials and Methods, Figures, Data Availability Section)
If your work benefited from core facilities, was their service mentioned in the acknowledgments section?	Not Applicable	

Design

Study protocol	Information included in the manuscript?	In which section is the information available? (Reagents and Tools Table, Materials and Methods, Figures, Data Availability Section)
If study protocol has been **pre-registered, provide DOI in the manuscript**. For clinical trials, provide the trial registration number **OR** cite DOI.	Not Applicable	
Report the **clinical trial registration number** (at ClinicalTrials.gov or equivalent), where applicable.	Not Applicable	

Laboratory protocol	Information included in the manuscript?	In which section is the information available? (Reagents and Tools Table, Materials and Methods, Figures, Data Availability Section)
Provide DOI OR other citation details if **external detailed step-by-step protocols** are available.	Not Applicable	

Experimental study design and statistics	Information included in the manuscript?	In which section is the information available? (Reagents and Tools Table, Materials and Methods, Figures, Data Availability Section)
Include a statement about **sample size** estimate even if no statistical methods were used.	Yes	A statement on sample size is included in the Method section.
Were any steps taken to minimize the effects of subjective bias when allocating animals/samples to treatment (e.g. **randomization procedure**)? If yes, have they been described?	Not Applicable	This study does not have randomization
Include a statement about **blinding** even if no blinding was done.	Yes	Laboratory technicians and scoring of the results was done blinded to the genetic basis of the samples
Describe **inclusion/exclusion criteria** if samples or animals were excluded from the analysis. Were the criteria pre-established?<br><br>If sample or data points were omitted from analysis, report if this was due to attrition or intentional exclusion and provide justification.	Not Applicable	All patients had a known genetic cause either at the beginning of the end of the study. Samples were only not included if a genetic cause was unknown.
For every figure, are **statistical tests** justified as appropriate? Do the data meet the assumptions of the tests (e.g., normal distribution)? Describe any methods used to assess it. Is there an estimate of variation within each group of data? Is the variance similar between the groups that are being statistically compared?	Yes	Statistical methods are described in the Methods section. Most of the results are descriptive. For the limited comparative analyses, normality of distribution was taken into account.

Sample definition and in-laboratory replication	Information included in the manuscript?	In which section is the information available? (Reagents and Tools Table, Materials and Methods, Figures, Data Availability Section)
In the figure legends: state number of times the experiment was **replicated** in laboratory.	Not Applicable	
In the figure legends: define whether data describe **technical or biological replicates**.	Not Applicable	

Ethics

Ethics	Information included in the manuscript?	In which section is the information available? (Reagents and Tools Table, Materials and Methods, Figures, Data Availability Section)
Studies involving **human participants**: State details of **authority granting ethics approval** (IRB or equivalent committee(s), provide reference number for approval.	Yes	The various sources with ethics approval are listed in the Methods section.
Studies involving **human participants**: Include a statement confirming that **informed consent** was obtained from all subjects and that the experiments conformed to the principles set out in the WMA Declaration of Helsinki and the Department of Health and Human Services Belmont Report.	Yes	For participants that have identification, informed consent was obtained (e.g. through the local study COMIRB 16-0146 or through the NAMDC study). For samples that were fully deidentified, the IRB did not require informed consent.
Studies involving **human participants: For** publication of **patient photos**, include a statement confirming that consent to publish was obtained.	Not Applicable	
Studies involving experimental **animals**: State details of **authority granting ethics approval** (IRB or equivalent committee(s), provide reference number for approval. Include a statement of compliance with ethical regulations.	Not Applicable	
Studies involving **specimen and field samples:** State if relevant **permits** obtained, provide details of authority approving study; if none were required, explain why.	Not Applicable	

Dual Use Research of Concern (DURC)	Information included in the manuscript?	In which section is the information available? (Reagents and Tools Table, Materials and Methods, Figures, Data Availability Section)
Could your study fall under dual use research restrictions? Please check biosecurity documents and list of **select agents and toxins** (CDC): https://www.selectagents.gov/sat/list.htm	Not Applicable	
If you used a select agent, is the security level of the lab appropriate and reported in the manuscript?	Not Applicable	
If a study is subject to dual use research of concern regulations, is the name of the **authority granting approval and reference number** for the regulatory approval provided in the manuscript?	Not Applicable	

Reporting

The MDAR framework recommends adoption of discipline-specific guidelines, established and endorsed through community initiatives. Journals have their own policy about requiring specific guidelines and recommendations to complement MDAR.

Adherence to community standards	Information included in the manuscript?	In which section is the information available? (Reagents and Tools Table, Materials and Methods, Figures, Data Availability Section)
State if relevant guidelines or checklists (e.g., **ICMJE, MIBBI, ARRIVE, PRISMA**) have been followed or provided.	Yes	A STROBE checklist is provided.
For **tumor marker prognostic studies,** we recommend that you follow the **REMARK** reporting guidelines (see link list at top right). See author guidelines, under 'Reporting Guidelines'. Please confirm you have followed these guidelines.	Not Applicable	
For **phase II and III randomized controlled trials**, please refer to the **CONSORT** flow diagram (see link list at top right) and submit the CONSORT checklist (see link list at top right) with your submission. See author guidelines, under 'Reporting Guidelines'. Please confirm you have submitted this list.	Not Applicable	

Data Availability

Data availability	Information included in the manuscript?	In which section is the information available? (Reagents and Tools Table, Materials and Methods, Figures, Data Availability Section)
Have **primary datasets** been deposited according to the journal's guidelines (see 'Data Deposition' section) and the respective accession numbers provided in the Data Availability Section?	Yes	Most of the primary data are available in EV2 and EV3 tables. Additional data are deposited in Zenodo and this information if provided in the manuscript.
Were **human clinical and genomic datasets** deposited in a public access-controlled repository in accordance to ethical obligations to the patients and to the applicable consent agreement?	Not Applicable	
Are **computational models** that are central and integral to a study available without restrictions in a machine-readable form? Were the relevant accession numbers or links provided?	Not Applicable	
If publicly available data were reused, provide the respective **data citations in the reference list**.	Not Applicable	
